# A commitment for *life:* Decades of unraveling the molecular mechanisms behind seed dormancy and germination

**DOI:** 10.1093/plcell/koad328

**Published:** 2024-01-12

**Authors:** Nikita Sajeev, Maarten Koornneef, Leónie Bentsink

**Affiliations:** Wageningen Seed Science Centre, Laboratory of Plant Physiology, Wageningen University, 6708PB Wageningen, the Netherlands; Laboratory of Genetics, Wageningen University, 6708PB Wageningen, the Netherlands; Max Planck Institute for Plant Breeding Research, Former Department of Plant Breeding and Genetics, Koeln 50829, Germany; Wageningen Seed Science Centre, Laboratory of Plant Physiology, Wageningen University, 6708PB Wageningen, the Netherlands

## Abstract

Seeds are unique time capsules that can switch between 2 complex and highly interlinked stages: seed dormancy and germination. Dormancy contributes to the survival of plants because it allows to delay germination to optimal conditions. The switch between dormancy and germination occurs in response to developmental and environmental cues. In this review we provide a comprehensive overview of studies that have helped to unravel the molecular mechanisms underlying dormancy and germination over the last decades. Genetic and physiological studies provided a strong foundation for this field of research and revealed the critical role of the plant hormones abscisic acid and gibberellins in the regulation of dormancy and germination, and later natural variation studies together with quantitative genetics identified previously unknown genetic components that control these processes. Omics technologies like transcriptome, proteome, and translatomics analysis allowed us to mechanistically dissect these processes and identify new components in the regulation of seed dormancy and germination.

## Introduction

**Figure 1.**
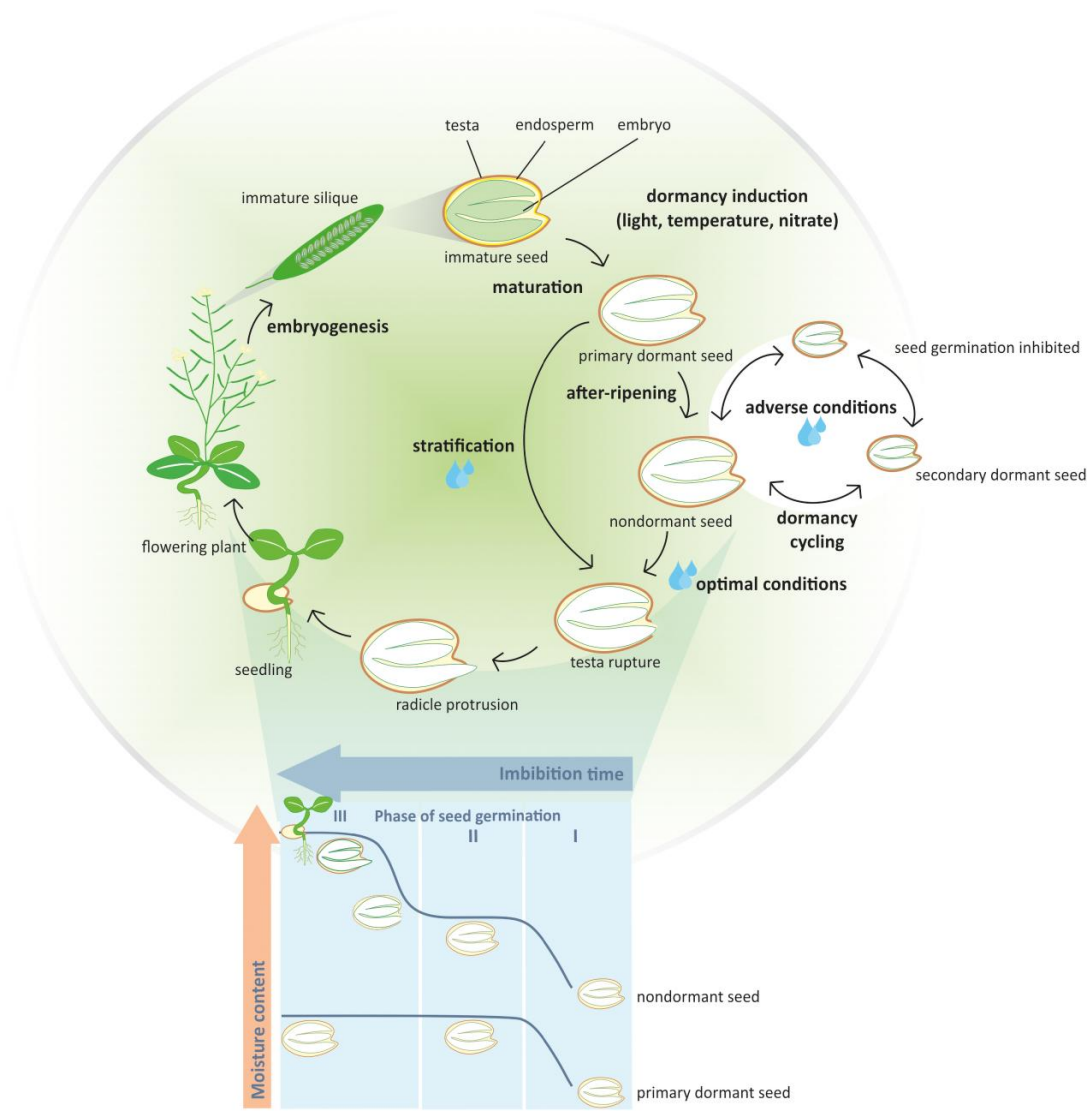
The life cycle of a seed. The seed life cycle begins with the embryogenesis phase occurring in the ovary of the flower of the mother plant. The ovary develops into a silique. Here the immature seed consisting of the testa, the endosperm and the embryo, which will form the future seedling, is developing. This stage is followed by seed maturation during which dormancy, desiccation tolerance, and seed longevity are induced. The induction of dormancy is dependent on genetic factors as well as environmental factors like light, temperature, and nitrate availability that the mother plant experiences. Seed maturation ends with a dry mature seed in which primary dormancy has been established. After-ripening or stratification can release dormancy and make the seeds nondormant. Germination of a nondormant seed can be inhibited, or the seed can reinter a dormant state if they are imbibed in adverse environmental conditions. If the nondormant seed is imbibed in optimal conditions, germination progresses through the different stages of germination, including TR and RP, which leads to the establishment of the seedling. Germination can be divided into 3 phases of water uptake. Stage 1 involves rapid uptake of water, which plateaus at stage II. Dormant seeds will remain in stage II and not progress to stage III of water uptake, whereas nondormant seeds will continue water uptake in stage III and complete germination. Imbibed stages indicated by the blue drops and the blue shades in the lower panel of the figure.

Seeds are complex entities that encompass all the genetic information needed to give rise to a new plant. Structurally, seeds are composed of 3 basic components: the embryo, which will form the future seedling; the energy-rich and nutritious tissue surrounding the embryo called the endosperm; and a protective layer on the outside called the testa or seed coat (Bewley et al. 2012) (Fig. 1). The seed coat also functions in producing protective metabolites, regulation of nutrient transport to the embryo, and defining the final seed size and shape (Francoz et al. 2018).

The process by which plants establish their next generation is called seed germination. This establishment is a primal goal for plants. Furthermore, seeds can travel for thousands of miles or wait for hundreds of years to germinate. This ability to remain in a quiescent state is called seed dormancy (Bewley 1997).

In the past decades numerous studies have been performed to unravel the mechanisms of seed dormancy and germination; however, we still know little about how emergence is inhibited in dormant seeds and how precisely the embryo emerges from the nondormant seed. Because reproduction is a crucial process in the plant life cycle, the genes regulating seed dormancy and germination are under enormous evolutionary selection pressure. It is challenging to measure dormancy and germination independently because this very much depends on the environmental context in which these measurements are made. An additional complication is that germination is often measured at a population level; when a batch of seeds germinates 100% in optimal conditions, the seeds are said to be nondormant.

In this review, we will attempt to untangle and shed light on the highly inter-linked seed dormancy and germination processes by reporting the most important scientific breakthroughs through time in this field. Here, we emphasize the use of genetic mutants, natural variation, and various omics studies that helped us understand the mechanisms and regulators of seed dormancy and germination. We will mostly focus on the model plant *Arabidopsis thaliana* because most comprehensive studies have been and are still performed with this species.

## The seed life cycle

The seed life cycle (Fig. 1) begins with the embryogenesis phase, in which a double fertilization event results in a single zygote. This zygote expands and grows in cell size and number to fill the embryo sac with the mature embryo (Bewley et al. 2012). The embryo growth is then arrested and followed by the maturation phase, in which the main goal is to accumulate resources and properties needed for successful germination and seedling establishment (Nonogaki et al. 2010). These resources can include mRNAs, sugars, and storage proteins required to kickstart germination. The maturation phase is also the critical phase wherein important traits like desiccation tolerance are established by intense moisture loss and seed dormancy and seed longevity are acquired. At the end of this phase, the seed is in a quiescent dry stage (Gutierrez et al. 2007; Holdsworth et al. 2008). Environmental conditions like light and temperature during the maturation phase, also termed as maternal or parental environment, are critical in determining the dormancy levels and the timing of germination in nature (Biere 1991; Fernandez-Pascual et al. 2013).

### Seed dormancy: a blessing in disguise

**Figure 2.**
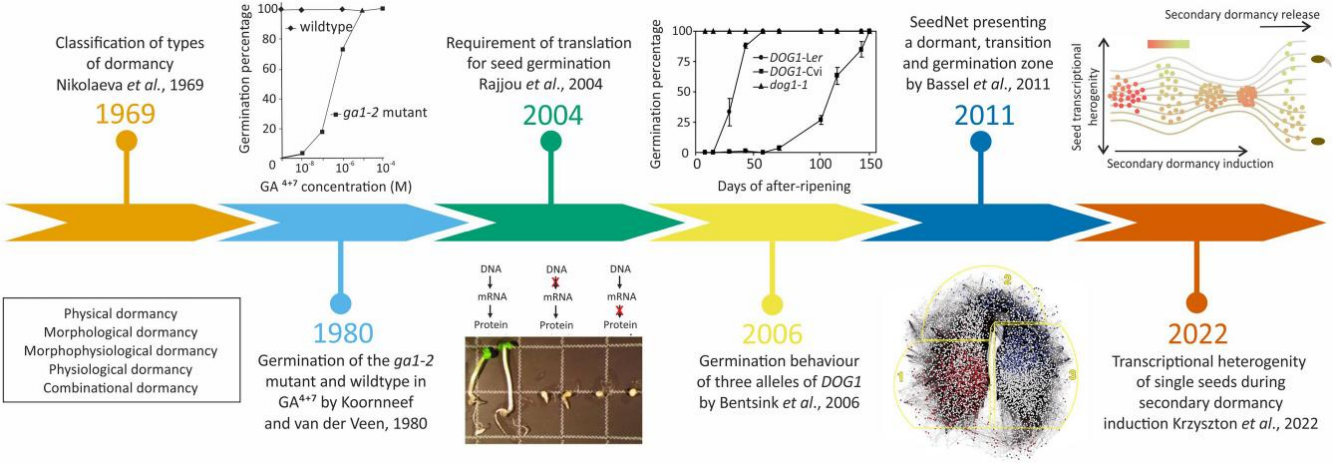
Milestones in the seed dormancy and germination research field through time.This figure graphically represents the biggest insights in the seed dormancy and germination field according to our interpretation. The first milestone was achieved in 1969, when Nikolaeva (1969) classified the 5 types of dormancies. This was followed by the breakthrough study of Koornneef and van der Veen (1980), where they concluded that the ABA concentration within the seeds determines the level of seed dormancy. The graph shows the germination percentage of GA-deficient mutants *ga1-2* and the wild-type seeds in increasing concentrations of exogenous GA. In 2004 it was discovered that the translation, but not transcription, is absolutely required for germination to occur (Rajjou et al. 2004). The next milestone arrived when Bentsink et al. (2006) cloned *DOG1*, the major quantitative trait locus governing seed dormancy in Arabidopsis and fine mapped it to the gene *DOG1 (*At5G45830). The graph here shows the germination percentages of 3 *DOG1* alleles Cvi, L*er*, and the mutant (*dog1-1*) in Arabidopsis during 0 to 150 days of AR. The graph shows the contribution of DOG1 to seed dormancy, as the *dog1-1* germinates 100% without a need for AR. In 2011, Bassel et al. generated a network model of global transcriptional interactions called SeedNet. This network represents 2 state-dependent sets of interactions that associate with dormancy and germination but also an intermediate transition region between these processes. The most recent milestone presented is from 2022, when Krzyszton et al. (2022) revealed transcriptional heterogeneity by performing RNA-seq on individual seeds during secondary dormancy induction. The figure shows that the heterogeneity is the lowest in very dormant seeds and increases when dormancy is broken.

Seed dormancy is defined as the inability of seeds to germinate in favorable conditions. Dormancy enables the mother plants to avoid risking germination of their entire progeny in an unpredictable and dynamic environment. Nikolaeva (1969) was the first seed biologist to develop a comprehensive classification scheme for the various types of seed dormancy. Dormancy can be broadly classified into 5 types of dormancies: physical, morphological, morphophysiological, physiological, and combinational dormancy (Fig. 2; Baskin and Baskin 2004). The most commonly studied type of dormancy is physiological dormancy. This type of dormancy is prevalent in gymnosperms and major classes of angiosperms, including Arabidopsis. The level of dormancy in seeds is highest at the end of the maturation phase and is called primary dormancy. Primary dormancy in Arabidopsis seeds can be released or alleviated by factors like after-ripening (AR; seed storage in the dry state) or stratification (cold or warm treatment in the imbibed state). The dormancy status also changes in response to environmental signals such as temperature, nitrate, and light quality exposure during seed development. Moreover, the sensitivity of the mature dry seeds to these signals depends on the AR time (Finch-Savage and Leubner-Metzger 2006; Nee et al. 2017a). If adverse environmental conditions prevail for extended periods of time, then after-ripened (AR) seeds can re-enter dormancy. This cycling of dormancy is known as secondary dormancy and can only occur when the seed is imbibed (Fig. 1) (Donohue 2005; Buijs 2020).

### Seed germination: the birth of a seedling

When nondormant seeds are imbibed in optimal conditions, seeds are “woken up,” so to say, and germination-promoting processes are initiated. By optimal conditions during imbibition of Arabidopsis seeds, we mean the presence of sufficient moisture, oxygen, optimal temperature, light quality, and absence of environmental stressors such as salinity, drought, cold, or heat stress. Regarding light quality, red light and its perception by photoreceptors called phytochromes is an essential factor for promoting seed germination (Longo et al. 2020). Germination itself is divided into 3 phases (Fig. 1). In Phase I there is tremendous uptake of water by the seed leading to swelling, followed by a phase wherein water uptake plateaus (Phase II). Dormant seeds will not surpass this stage, whereas nondormant seeds will move on to Phase III, in which water uptake is resumed and the seed coat or the testa will rupture (TR). This is a critical physiologically visible stage during seed germination. After TR, cell elongation and expansion take place, which will eventually lead to the embryonic root or radicle to protrude through the endosperm (radicle protrusion [RP]). Once RP has occurred, germination is said to be complete (germination “sensu stricto”). The phase between TR and RP is a very sensitive one because the commitment to germinate into a seedling cannot be reversed once RP has occurred. This could be because seeds drastically lose their desiccation tolerance ability upon RP (Maia et al. 2011). Hence, nondormant seeds can stop or slow germination before RP if environmental conditions do support successful seedling establishment. Therefore, the final commitment to germination is made by integrating signals from the environment, and this process is tightly regulated at the genetic level.

## Hormonal control of seed germination and dormancy

**Figure 3.**
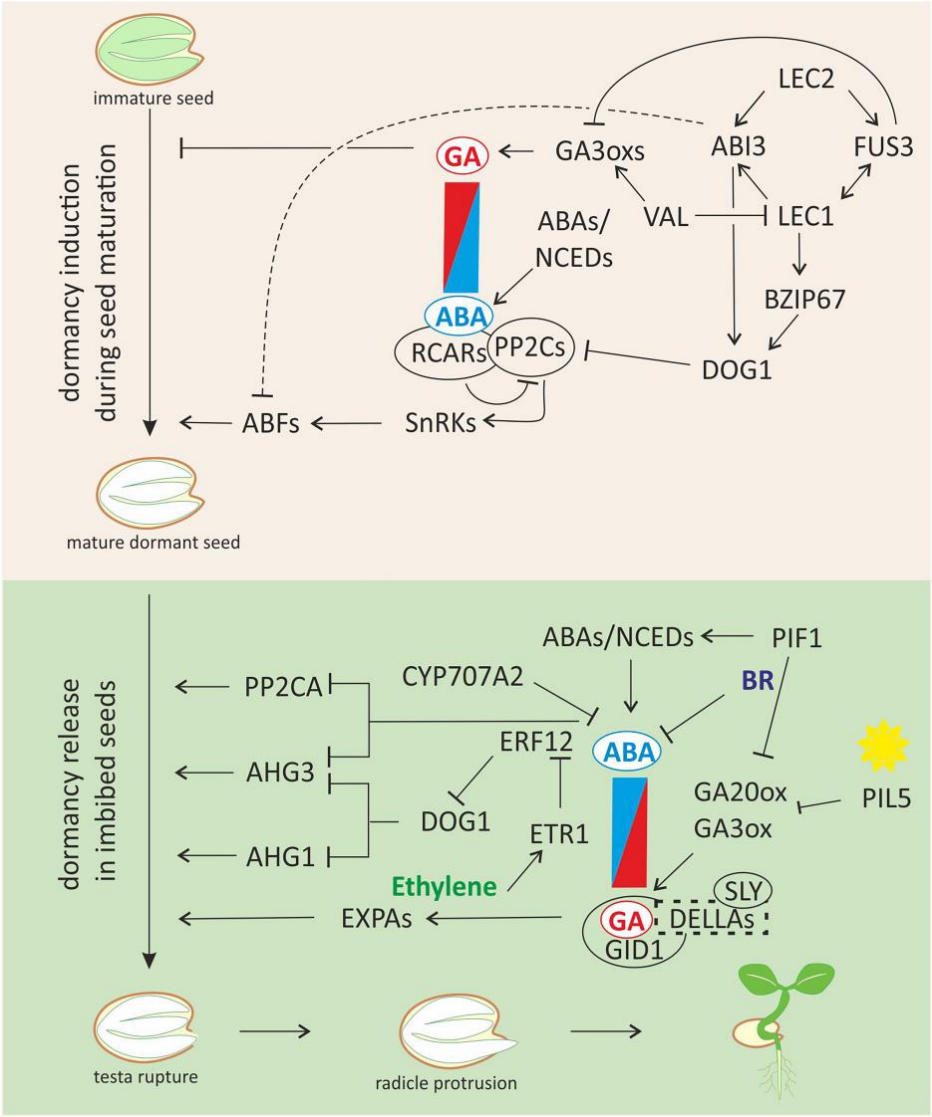
The regulation of seed dormancy and germination during seed maturation and dormancy release in imbibed seeds. The figure represents a schematic overview of gene interactions during induction of seed dormancy in the immature seeds and during dormancy release in the imbibed state. Key for both stages is the balance between concentrations of GA and ABA, indicated by the red/blue rectangle. Both hormones are sensed during seeds maturation and dormancy release; however, in the scheme, factors involved in the perception of ABA (RCARs and PP2Cs) are indicated in more detail the upperpart of the figure, which represents factors involved in dormancy induction during seed maturation. Those involved in GA perception (GID1, DELLAs, and SLY) are depicted in the lower panel, which shows the interactions during the dormancy release in the imbibed seeds. Arrows indicate a promotive effect, bars a repressive effect. The line between ABI3 and ABFs is dashed because the role of ABI3 in ABA perception has remained unclear so far. The indicated interactions are also described in the main text.

The absolute requirement of abscisic acid (ABA) and gibberellins (GAs) for the regulation of seed dormancy and germination was revealed in Arabidopsis and tomato mutants defective in the biosynthesis of these hormones in the early 1980s (Fig. 2) (Koornneef and van der Veen 1980; Groot and Karssen 1987). Most of these mutants were initially isolated on the basis of their altered seed germination phenotype. The requirement of GA for germination depends on the ABA concentration within seeds, as could be concluded from the observation that GA-deficient mutants can germinate in the absence of ABA. The latter led to the isolation of ABA-deficient mutants as revertants of GA-deficient mutants, which can germinate without GA or on high concentrations of the GA biosynthesis inhibitor paclobutrazol (Koornneef et al. 1982; Léon-Kloosterziel et al. 1996). The biosynthesis mutants (especially those in Arabidopsis, maize, and rice) were crucial in elucidating the biochemical steps and genes of the biosynthetic pathways for both plant hormones. For this the chemical analysis of the mutants was important. The cloning of the underlying genes was based on the mutants using map-based cloning and transposon tagging, also described as forward genetics. In the meantime, the signal transduction steps were also identified. Due to the fact that many of these steps are controlled by multiple genes, this required additional procedures such as chemical genetics and biochemical interaction studies [reviewed by Cutler et al. (2010)]. At this stage, increasing use was made of reverse genetics of candidate genes and the characterization of these mutants and double mutants with seed dormancy tests. Overall, the regulation of seed dormancy and germination and especially the role of ABA and GA in this has been extensively reviewed (Finch-Savage and Leubner-Metzger 2006; Finkelstein et al. 2008; Holdsworth et al. 2008; Bewley et al. 2012; Graeber et al. 2012; Shu et al. 2016). Below we aim at providing an overview of the essential roles of these 2 hormones. Gene interactions during the induction of seed dormancy in immature seeds and during dormancy release in the imbibed state are schematically presented in Fig. 3.

### ABA, a repressor of germination and inducer of seed dormancy

In addition to not needing GAs for germination, ABA-deficient *aba1* mutants showed an absence of AR requirement in the shallow dormant Arabidopsis accessions Landsberg *erecta* (L*er*) and Columbia (Col; Koornneef et al. 1982). Severe ABA biosynthesis mutants (*aba1*, *aba2*, *aba3*, and the *nced6/9* double mutant) show an absolute lack of seed dormancy (Lefebvre et al. 2006). The ABA concentration in seeds is, in addition to the control of ABA biosynthesis, also determined by conversion of ABA into phasic acid leading to inactivation of ABA. Responsible for this conversion are the ABA-8’ hydroxylase(s), which are 2 P450 cytochromes encoded by CYP707A1 and A2. These *cyp707a* mutants have an increased dormancy phenotype (Kushiro et al. 2004).

ABA levels in mature seeds are the result of synthesis during seed maturation. Depending on the species, the ABA levels peak at 2 moments (Kozaki and Aoyanagi 2022). Detailed analysis of ABA concentrations during imbibition showed that although ABA levels initially decrease, they increase at later imbibition stages in dormant seeds (Ali-Rachedi et al. 2004). Moreover, it is ABA produced and released by the endosperm that represses embryo germination, as was revealed by a “seed coat bedding” assay (Lee et al. 2010). The expression of the DELLA protein RGL2 is required for this response. The importance of ABA in maintaining the dormant state in imbibed seeds is also confirmed by the germination-promoting effect of the ABA biosynthesis inhibitor fluoridone when applied during the imbibition of dormant seeds. ABA in seeds is mainly synthesized in the endosperm and transported to the embryo by 4 ATP-binding cassette (ABC) transporter genes (Kang et al. 2015). AtABCG25 and AtABCG31 export ABA from the endosperm, whereas AtABCG30 and AtABCG40 import ABA into the embryo (Kang et al. 2015). The role of ABA transported from the mother plant during dormancy induction is minor, as was shown by ABA-deficient mother plants with ABA-producing embryos and endosperms obtained by reciprocal crossing of *aba1* mutants with its wild type (Karssen et al. 1983).

### Seed-specific ABA signaling

The complex regulation of the ABA signaling pathway in seeds, with many gene copies present for its major steps, has recently been described by Née and Krüger (2023). Mutant screens for seeds germinating in the presence of wild type inhibiting concentrations of ABA identified several genes, among which were ABA insensitive 3 (*ABI3*), *ABI4*, and *ABI5*. These genes are important seed-specific downstream ABA targets and are considered repressors of germination (Koornneef et al. 1984; Finkelstein 1994). Based on their specific expression during seed development and the early stages of germination, they are considered to have seed-specific roles.

Studies using strong *ABI3* mutants revealed, in addition to a lack of dormancy, many important defects during seed development, including the absence of chlorophyll degradation and desiccation intolerance (Ooms et al. 1993; Nambara et al. 1994). The *ABI3* gene together with the seed maturation genes *LEAFY COTYLEDON 1* and *2* (*LEC1/2*) and *FUSCA3* (*FUS3*) form the LAFL (*LEC1*, *ABI3*, *FUS3*, *LEC2*) regulatory network (Carbonero et al. 2017). These genes are essential for seed maturation, and single gene mutants show partial overlap in their phenotypes (Raz et al. 2001; Jia et al. 2013). All these genes (except *LEC1*) are members of the AFL (ABI3/FUS3/LEC2) subfamily of B3 transcription factors (TFs) and bind G-box like sequences in the promoters of seed maturation genes, which are bound by basic leucine zipper (bZIP) TFs (Boulard et al. 2017; Carbonero et al. 2017). ABI3 has many direct targets, including ABI5, DELLAs, and the bHLH type TF PIL5 (PHYTOCHROME INTERACTING FACTOR-LIKE 5) (Tian et al. 2020). ABI3, ABI5, and DELLAs together activate the expression of SOMNUS, which encodes a C3H type zinc finger protein that regulates the expression of genes involved in the synthesis of ABA and in the catabolism of GA in imbibed seeds, thus inhibiting seed germination (Park et al. 2011; Lim et al. 2013). The molecular interaction of ABI3 with the primary ABA signaling pathway remains unclear and is based only on the extreme ABA insensitivity of its mutant. The latter is not observed for the *fus3* mutants despite that they displayed a similar absence of dormancy as the *abi3-3* mutant (Keith et al. 1994).

*ABI4* and *ABI5* were also identified in a mutant screen for ABA-insensitive mutants and thereby seemed also to be candidates in the ABA signal transduction (Finkelstein 1994). Their mutant phenotypes were much less severe than that of *abi3* mutants; however, they showed a strong reduction of ABA/ABI3-induced genes, such as the downregulation of the LATE EMBRYO ABUNDANT proteins *AtEM1* and *AtEM6* gene expression (Finkelstein 1994; Carles et al. 2002). Conversely, the *abi4* and *5* mutants did not show clear seed dormancy phenotypes. The *ABI4* gene, reviewed by Chandrasekaran et al. (2020), encodes an APETALA2/ethylene response factor type TF that affects many processes by regulating gene expression, including that of the ABA degradation genes ABA 8’ hydroxylase(s) (*Cyp707A1* and *Cyp707A2*), as well as genes involved in sugar signaling. Additionally, it downregulates several GA biosynthesis genes. The *abi4* mutant has a small effect on the rate of germination, and the mutant is not affected in its seed development phenotype (Shu et al. 2013).

ABI5, reviewed by Skubacz et al. (2016), encodes a bZIP TF, which can both bind to the ABA Responsive Element in promotors and interact with ABI3 via its B1 domain. The protein also interacts with AFPs (ABA 5 binding proteins), which allows interaction with other proteins (Lynch et al. 2017). Overexpression of AFP1 and AFP2 resulted in extreme ABA tolerance and desiccation-intolerant seeds resembling strong *abi3* mutant alleles and suggests that these genes are major actors in seed-specific ABA signaling. Deng et al. (2023) demonstrated that AFP2 negatively regulates primary seed dormancy by suppressing the expression of the major regulator of seed dormancy, *DELAY OF GERMINATION 1* (*DOG1*). The TF WRKY36 can interact with AFP2 to transcriptionally co-repress TOPLESS RELATED PROTEIN 2 and epigenetically silence *DOG1* by reducing histone acetylation. The *abi5* mutants themselves show tolerance to germination inhibition by NaCl and osmotic stress (Carles et al. 2002). ABI5 can be phosphorylated by SnRK2 proteins as part of the ABA signal transduction chain. The phosphorylation of ABI5 enables interactions with other proteins, including EM1 promotors, suggesting a role in the acquisition of desiccation tolerance in seeds. Furthermore, ABI5 can interact with other hormones, including GA, jasmonic acid, and auxin pathways (Skubacz et al. 2016). Overall, it seems that ABI5 is crucial for part of the ABA transduction chain in seeds, early germination, and its response to stresses.

### GA promoters of seed germination

GAs are essential for germination because GA biosynthesis mutants such as *ga1/ga2/ga3* require application of exogenous GAs for germination (Fig. 2, Koornneef and van der Veen 1980). The GA requirement for germination is further indicated by the observation that GA biosynthesis inhibitors such as paclobutrazol and unicazol inhibit germination. GA biosynthesis and GA signaling in seeds has been reviewed by Urbanova and Leubner-Metzger (2018). Active GA binds to the receptor GID1 (encoded by *AtGID1a*, *AtGID1b*, and *AtGID1c*), and together they repress the DELLA proteins. The latter are repressors of GA signaling, and especially RGA2 is the seed-specific representative of this group of 5 proteins (Lee et al. 2002).

The role of GA seems mainly to overcome restraints that are partially related to (ABA induced) dormancy but also to overcome the resistance of the embryo surrounding tissues, including that conferred by the integuments (Debeaujon and Koornneef 2000) and the endosperm (Groot et al. 1988). The latter aspect relates to the induction of genes encoding cell wall–degrading enzymes by GA [reviewed by Nonogaki (2019)]. Focusing on expansin mediate cell-wall loosening, Xu et al. (2020) elucidated the pathway by which DELLA proteins and nitric oxide (NO) control TFs (including TCP14/15, RAP2.2/2.3/2.12, and ZML1), which in turn control expansin gene expression.

### Hormonal crosstalk during dormancy and germination

ABA and GA do not regulate seed dormancy and germination on their own. Where interactions with jasmonic acid and auxin were already mentioned, brassinolides (BRs) and ethylene are also known to affect these traits. BRs stimulate germination by promoting TR and endosperm rupture in Arabidopsis in a similar way as GAs do. BRs can rescue the germination phenotypes of the GA biosynthetic mutant *ga1-3* and the GA-insensitive mutant *sleepy1* (Steber and McCourt 2001). Germination does not entirely depend on GA response but rather on GA and BR working in parallel. Two key basic helix–loop–helix transcription factors in the BR signaling pathway, BRASSINOSTEROID ENHANCED EXPRESSION 2 (BEE2) and BEE 2 INTERACTING with INCREASED LEAF INCLINATION 1 BINDING bHLH1 (HBI1), are involved in the regulation of endosperm rupture. Expression of BEE2 and HBI1 was induced in response to BR and GA treatment (Zhong et al. 2021). Furthermore, BRI1-EMS-SUPPRESSOR 1 (BES1) affecting BR signaling was shown to physically interact with ABI5 to hinder its DNA binding capacity, attenuating the ABA-mediated suppression of seed germination by lowering the expression of ABI5 targets. BIN2, a repressor of BR signaling, promotes the ABA responses (Zhao et al. 2019). BES1 and DELLAs were reported to mediate the crosstalk between BRs and GAs during cell elongation in Arabidopsis [reviewed by Ross and Quittenden (2016)].

Ethylene is another promoter of germination. The loss-of-function mutant of one of the ethylene receptors ETHYLENE RESPONSE FACTOR 1 (ETR1) was identified by its reduced dormancy phenotype and therefore named (*reduced dormancy 3;* Li et al. 2019). It was shown that ETR1 represses the expression of *ERF12*, which recruits TOPLESS to form a repressor complex and binds to the DOG1 promoter, thereby inhibiting the expression of *DOG1* and leading to reduced seed dormancy by ethylene.

Another mechanism controlled by EIN2 (ETHYLENE INSENSITIVE 2), a component of ethylene signal transduction, was recently described by Guo et al. (2023). The authors concluded that EIN2 can function independently from the canonical ethylene pathway acting via EIN3/EIL1, by inhibition of a histone H3 acetylase encoded by *HLS1* (*HOOKLESS1*). When active, HLS1 acetylates H3 histones in the region of *ABI5* and *ABI3*, which represses their transcription. In the absence of EIN2, the expression of these ABA signaling molecules is upregulated and germination is inhibited.

Auxin has been shown to function both positively and negatively in seed germination depending on its dose. It has also been reported that high doses of auxins enhance seed dormancy by activating ABI3 mediated by the AUXIN RESPONSE FACTOR 10 TF (Liu et al. 2013) and IAA8 (Hussain et al. 2020). This leads to enhanced expression of *ABI3*, although not by binding to the *ABI3* promotor. *IAA8* expression is upregulated by cold and by ROS signaling and was found to associate with the ABI3 promotor (Hussain et al. 2020). In both cases the auxin effects were relatively minor in affecting the rate of germination. Wang et al. (2016) emphasized the specific role of auxin in the RP and thereby germination rate. They noticed that this is, to a large extent, controlled by the auxin transporter AUX1. *AUX1* expression is negatively controlled by the histone acetylation genes *SNL1/SNL2*, which decrease in expression upon after dormancy breakage.

## The power of natural variation, among others, a DOG1 story

Seed germination is a quantitative trait for which ample genetic variation is present in nature. Especially seed dormancy, which together with the timing of flower initiation is a main factor determining where and when a seed should germinate, has shown to be a key trait defining local adaptation. In European accessions a North-South cline was identified, where (primary) dormancy is lower in the North compared with the South. A similar pattern was found for accessions collected in the Iberian Peninsula (Debieu et al. 2013; Vidigal et al. 2016). Studies on the Iberian population also show that dormancy is generally lower at higher altitudes. Kronholm et al. (2012) described genetic signatures of local adaptation as well as a negative correlation between dormancy and summer precipitation. These clines are likely explained by the shorter growing season (in the Northern regions and higher altitudes) and the riskier environment with a lower chance of survival in the Southern regions and lower altitudes. The importance of the right dormancy levels is illustrated in the work of Huang et al. (2010). They showed with a garden experiment that genetic variation for fitness and germination phenology are associated with the genetic regions that were earlier detected for primary seed dormancy by testing AR requirement in the laboratory.

The use of natural variation for the identification of seed dormancy loci has led to the identification of the key regulator of seed dormancy, *DOG1*. Natural variation for dormancy was first explored in the L*er* Col recombinant population (RIL) (Van der Schaar et al. 1997). However, the use of both these accessions with low dormancy levels only revealed minor quantitative trait loci (QTL). A breakthrough was made when more dormant accessions were used for these genetic approaches. The L*er*, Cape Verde Island (Cvi) RIL population yielded 7 QTL that together explained more than 60% of the total variance (Alonso-Blanco et al. 2003). The main QTL identified in this study was the aforementioned *DOG1* QTL. Experiments with different RIL populations mainly resulted in the identification of the already known DOG loci (Meng et al. 2008; Bentsink et al. 2010; Amiguet-Vercher et al. 2015; Footitt et al. 2020). Moreover, genome-wide association studies using Arabidopsis accessions from all over the world also identified the already known QTL (He 2014; Martínez-Berdeja et al. 2020), sometimes with a few additional loci (Chen et al. 2023).

So far we have been discussing mostly natural variation for seed dormancy, measured as AR requirement. However, there is also genetic variation for germinability in cold and dark (Meng et al. 2008) and germination variability (Abley et al. 2021), which is referred to as the heterogeneity in germination that occurs in genetically identical plants that have been grown in the same condition. The level of germination variability differs between Arabidopsis accessions, and QTL analyses using MAGIC lines revealed QTL in the *DOG1* and *DOG6* regions (Abley et al. 2021). The major Cold-tolerant Dark Germination QTL occurred at positions for which no QTL have been cloned so far (Meng et al. 2008).

### Identifying the genes underlying the DOGs: an ongoing search

The identified *DOG* QTL underly genes of a different nature; so far *DOG1*, *DOG6*, and *DOG18* are cloned, and these encode, respectively, an unknown protein, a NAC transcription factor, and a pseudo phosphatase (RDO5) (Bentsink et al. 2006; Xiang et al. 2016; Song et al. 2021). Although we can only speculate about the nature of the remaining *DOG* loci, genetic analyses and transcriptional profiling have indicated that they control dormancy by different additive genetic and molecular pathways (Bentsink et al. 2010). Recently, several reviews have appeared that describe the regulation of dormancy via DOG1 (Nee et al. 2017a; Carrillo-Barral et al. 2020; Li et al. 2020; Soppe and Bentsink 2020; Iwasaki et al. 2022). *DOG1* was identified using a combined genetic approach involving the fine-mapping of the identified QTL region and a mutagenesis analyses in the background on the near isogenic line (NIL) that contained the *DOG1* Cvi allele (Bentsink et al. 2006). The nondormant mutant identified in the NIL background could not be genetically separated from the *DOG1* dormant allele and turned out to be a mutation in the DOG1 gene. Knowing that knocking-out DOG1 would lead to loss of dormancy allowed for screening knockout lines in the fine-mapped region of the QTL. As such, At5G45830, a gene of unknown function, was identified as the gene encoding *DOG1*. The availability of 3 alleles (Cvi, L*er*, and the mutant *dog1-1*), which showed very strong dormancy phenotypes, was of great importance for the later studies (Fig. 2; Bentsink et al. 2006). It is the amount of DOG1 protein that determines the level of seed dormancy, and loss of function of the protein that occurs during AR leads to a reduction of seed dormancy (Nakabayashi et al. 2012). DOG1 interacts with clade A PP2C phosphatases, the ABA-HYPERSENSITIVE GERMINATION 1 and 3 (AHG1 and 3). DOG1 likely suppresses the action of AHG1 and 3 and as such inhibits the release of seed dormancy (Nee et al. 2017b).

There is a clear link between ABA and DOG1, which involves the above described interaction with the AHG proteins but is also based on the fact that DOG1 cannot confer dormancy in a ABA-deficient/-insensitive background (Bentsink et al. 2006). The fact that DOG1 is an α-helical heme-binding protein is another link to ABA (Nishimura et al. 2018). Heme binding proteins, which are reported to function as sensors for oxygen and NO, are required to counteract ABA during dormancy release (Arc et al. 2013). DOG1 is post-translationally modified as the result of AR (Nakabayashi et al. 2012). It is hypothesized that DOG1-bound heme could cause non-enzymatic oxidative cellular damage by promoting ROS formation during seed dry storage; this would be more severe in the presence of larger amounts of DOG1 protein (Chiabrando et al. 2014).

DOG1 expression is induced during seed maturation (Bentsink et al. 2006). In-depth analyses revealed that during seed development, DOG1 genetically interacts with ABI3 and as such affects other seed maturation traits as well (Dekkers et al. 2016b). Seeds of the *dog1* mutant have reduced expression of genes encoding LATE EMBRYOGENESIS ABUNDANT and HEAT SHOCK PROTEINs and contain reduced amounts of certain primary metabolites and oligosaccharides. Interestingly, the *abi3-1 dog1-1* double mutant has green seeds, which phenocopies the more severe *abi3* mutants. Based on sequence alignment, DOG1 belongs to a family of 6 members, the DOG1-Like (DOGL) genes. None of these proteins affect seed dormancy; however, DOGL4 strongly induced the expression of seed maturation–specific genes, including the major seed reserve proteins ALBUMIN, CRUCIFERIN, and OLEOSIN (Sall et al. 2019).

The regulation of *DOG1* has intensively been studied. Lower temperatures during seed maturation led to increased levels of DOG1 transcript and protein (Nakabayashi et al. 2012). This effect is mediated by an increased expression of the bZIP transcription factor bZIP67, which transactivates *DOG1* expression. Genetic variation in the bZIP67 binding sites of the DOG1 promoter provides a mechanism to explain natural genetic variation in *DOG1* expression (Bryant et al. 2019). Moreover, low temperatures, when perceived during seed maturation, are known to increase DOG1 expression, as well as dormancy (Nakabayashi et al. 2012; He et al. 2014). Gene-swapping experiments between Arabidopsis and the nondormant *Lepidium sativa* showed that the DOG1 function is conserved among these Brassicaceae (Graeber et al. 2014). Whereas biomechanical analyses that allow to investigate the material properties of the endosperm so far have never left the pilot phase in Arabidopsis, these are common ground in *Lepidium* (Muller et al. 2006; Voegele et al. 2012). Such analyses reveal that DOG1 inhibits the expression of cell-wall remodeling genes via temperature-dependent adjustments of key enzymes of the GA biosynthetic pathway (Graeber et al. 2014). How a role for *DOG1* in the endosperm aligns with *DOG1* expression in the embryo remains unknown.

In addition to external factors regulating the expression of DOG1, several mechanisms that involve the DOG1 gene structure have been identified to regulate DOG1 expression. These include the following: 1) alternative splicing and selective polyadenylation resulting in short DOG1 and long DOG1 isoforms (Cyrek et al. 2016; Fedak et al. 2016); 2) long noncoding RNAs in the 5’ region of the gene, which are referred to as PUPPIES and promote DOG1 expression in high-salt conditions (Montez et al. 2023), and an antisense RNA located in the 3’ region of the gene referred to as *1GOD* that negatively affects *DOG1* expression (Cyrek et al. 2016; Kowalczyk et al. 2017); and 3) epigenetic regulation as summarized by Ding et al. (2022).

*REDUCED DORMANCY 5* (*RDO5*), originally identified in the same genetic screen as the *dog1-1* mutant and underlying the natural variation at the *DOG18* locus (Xiang et al. 2016), has also been identified to bind to DOG1. RDO5 is annotated as a so-called pseudo phosphatase; although it belongs to the PP2C phosphatase family, it lacks phosphatase activity (Xiang et al. 2014). RDO5, similarly to DOG1, is localized to the nucleus. Three members of the PUM family of RNA binding proteins were upregulated in the *rdo5* mutant, suggesting that these genes promote germination. The PUM proteins are, in contrast to DOG1 or RDO5, localized in the cytoplasm; therefore the regulation of *PUM*s by RDO5 is indirect. In agreement with a role for PUMs in the translational regulation of seed germination, these might be downstream factors that regulate the onset of germination and not dormancy specifically. Downregulation of PUM9 and 11 by RNAi in the *rdo5* mutant background resulted in increased dormancy levels. PUMs are RNA binding proteins and are therefore discussed in more detail in the section “Mechanisms underlying translational control: The role of RNA binding proteins.”

*DOG6* encodes the NAC transcription factor *ANAC60* (Song et al. 2021, 2022). Different natural alleles of *DOG6* determine the localization of the protein due to the presence or absence of a membrane binding domain. Absence of the membrane binding domain allows the protein to enter the nucleus and as such attenuate seed dormancy.

## Exploring transcriptome profiles

Transcriptome analyses have been a preferred method to address seed dormancy­– and seed germination–related questions. In the early days, transcript profiling was based on hybridizing mRNAs to cDNA libraries that were constructed from specific tissues. One of the first reports revealing transcriptional changes during seed germination was that of Harada et al. (1988), who made use of nitrocellulose arrays containing a seedling cDNA library. These authors reported on the temporal and spatial specificity of these transcripts in *Brassica napus* cotyledons and seedling axes based on hybridization analyses of the identified clones. The nitrocellulose carriers used for hybridization of mRNA to cDNAs were replaced for coated slides in 1995 and around 2015 for RNA sequencing (RNA-seq). Before the switch to microarrays was made, Bove et al. (2005) reported a cDNA-AFLP study during dormancy breaking in *Nicotiana plumbaginifolia* seeds. Of the 15,000 cDNA fragments identified, 1,020 responded differentially to dormancy breaking or maintenance. The first seed germination–related study involving gene expression analysis using microarrays also dates from 2005 (Nakabayashi et al. 2005). This work performed on Arabidopsis seeds led to the identification of 12,470 seed-stored mRNAs. More than 10,000 of these transcripts were also detected at the different imbibition time-points investigated (6, 12, and 24 hours after imbibition). Differential expressed genes include those related to metabolism, transcription, cell cycle, and DNA processing. Moreover, the analyses of seeds at different stages during imbibition revealed multiple regulatory mechanisms, including epigenetic chromatin structures, co-regulated gene clusters, and cis-acting elements. The first RNA-seq study on seeds revealed 50% more differentially expressed genes then initially reported by Nakabayashi et al. (2005) based on microarray analyses (Narsai et al. 2017). In this section we will discuss transcriptome analyses in dormant and germinating seeds and focus mostly on genome-wide analyses.

### Differences between dormant and nondormant seed transcriptomes

Seeds are full of stored mRNAs for which translation is considered important for germination to occur. The importance of stored transcripts for seed germination raised questions about the changes in the transcriptome, particularly when seeds transition from the dormant to the nondormant state during seed AR (dry storage). Although it has been debated whether transcriptional changes, especially upregulation, can occur in dry seeds, studies supporting such findings have been reported for *N. plumbaginifolia*, barley, and Arabidopsis seeds (Bove et al. 2005; Finch-Savage et al. 2007; Leymarie et al. 2007). More specifically, it has been suggested that specific mRNAs are being oxidized during AR. In sunflower seeds 24 oxidized transcripts have been identified that belong to the gene ontology classes metabolic processes, response to stress, and transport. Specific genes identified include protein phosphatase 2C PPH1, mitogen-activated protein kinase phosphatase 1, and phenyl ammonia lyase 1 (Bazin et al. 2011).

More obvious dormancy-specific transcriptomes can be revealed when seeds are investigated in the imbibed state. The comparison of dormant and AR Cvi seeds revealed an upregulation of genes related to translation, including genes that encode ribosomal proteins, translation initiation, and elongation factors in AR seeds (Cadman et al. 2006). This suggests that translation is turned down in dormant seeds, which has been confirmed by the analyses of translation by means of polysome profiling. In imbibed dormant seeds, translation starts like it does in AR seeds; however, it does not reach the same level as it does in nondormant seeds (Bai et al. 2018). Another attempt to identify differences between dormant and AR seeds revealed a set of 30 genes with similar expression profiles in the different physiological stages investigated; among these genes was the key dormancy regulator *DOG1* (Finch-Savage et al. 2007). Although all these 30 genes in theory have the potential to be regulators of seed dormancy or seed germination, they are not reported as such. Only ERF1, which was associated with temperature-sensitive germination in lettuce and improved germination in saline conditions in rice, was described to be germination related (Yoong et al. 2016).

Investigating the temporal changes in dormant and nondormant seeds revealed that transcriptional patterns already differ at 12 hours of imbibition, when nondormant seeds prepare for germination (Dekkers et al. 2016a). After the initial imbibition, which leads to large transcriptional changes (up and down) in both dormant and nondormant seeds (approximately 1,000 and 2,000, respectively), most changes occur in nondormant seeds. Up to 2,000 transcripts change when comparing each time point with the previous, whereas in the dormant seeds this is limited to less than 100. The transcriptional profiles that discriminate dormant and nondormant seeds are found both in the embryo and endosperm and show great overlap with transcriptional changes in seeds in the soil seed bank that cycle in and out of dormancy, suggesting that laboratory-based experiments are a good alternative to study natural processes (Buijs et al. 2020). Transcriptional differences between physiological stages can be used to reveal genes that regulate processes of interest. An example of this is a reverse genetics approach that was chosen to identify the role of genes that are differentially expressed in 24-hour imbibed dormant and AR seeds of 5 different genotypes (L*er* and 4 *DOG* NILs) (Yazdanpanah et al. 2017). Analyses in imbibed dormant and AR seeds in the same 5 genotypes revealed that dormancy maintenance and germination are likely to be very conserved processes (Bentsink et al. 2010). Of the total 3,689 differentially expressed genes, 1,720 were dormancy and 1,969 germination upregulated; of these 45 and 25 were common between all genotypes. Among these genes were the earlier identified germination related GA3OX2, PIF6, and ASG5. Moreover, for 50% of the genes for which T-DNA mutants were available, a germination phenotype could be observed.

### Insights from network analyses

The initial transcriptome analyses did not yield new key regulators of seed dormancy and germination, and this became a reason for Bassel et al. (2011) to perform a coexpression analysis on publicly available expression data from imbibed mature seeds. The condition-dependent network SeedNet (http://netvis.ico2s.org/dev/seednet/#/) consisted of 1,583 transcripts associated with germination and 1,844 transcripts associated with nongermination (Fig. 2). The germination and nongermination subdomains were connected by a domain referred to as the intermediate transition region, which was enriched for genes involved in cellular phase transitions, including *SERRATE* and *EARLY FLOWERING SHORT DAYS*. Knockouts of the latter show both a flowering time and germination phenotype (Soppe et al. 1999). Overall, this work shows that network studies not only have the potential to identify co-regulated genes but also possible phase transitions.

Transcriptional phase transitions during seed germination were also identified by (Dekkers et al. 2013), who investigated spatial and temporal transcriptome changes during seed germination. Arabidopsis seeds were dissected into 4 sections: cotyledons, radicle and hypocotyl, micropylar and chalazal endosperm, and peripheral endosperm. These tissues were investigated from the start of seed imbibition until endosperm rupture at 10 time-points, of which at TR (25 HAI) and endosperm rupture (38 HAI), the transcriptomes of ruptured and nonruptured seeds had been separated. Whereas at 25 hours after imbibition (HAI) the transcriptome of the nonruptured seeds do not differ from those at 20 HAI, there are a large number of differential expressed genes, mostly upregulated, in the TR seeds compared with the nonruptured in both the embryo and endosperm tissues. These transcriptional changes separate germination into 2 transcriptional phases: before and after TR. The transcriptional changes over time have been captured by a coexpression network (Dekkers et al. 2013). The latter showed that coexpression networks form highly confident templates for hypothesis generation, which as such can be further scrutinized to unravel molecular networks. An example of this is the interaction mapping study performed for the cell wall–loosening EXPANSIN (EXPA) gene family (Xu et al. 2020). The EXPA gene family is associated with cell wall modification, and the expression of several family members is induced during Arabidopsis seed germination. One of these expansins, *EXPA9* is regulated by the transcription factors *TCP14* and *TCP15*. Xu et al. (2020) show that the reduced germination phenotype of the *tcp14 tcp15* double mutant can be partially overcome by EXPA9 ectopic expression.

### New developments in transcriptome analyses

Recently, we have entered a new era of transcriptome analyses: single-cell sequencing. For seeds this is not yet straightforward; however, a first attempt that includes imbibed and germinated seeds has recently been presented (Lee et al. 2023). In single-cell sequence analyses, cell types are annotated based on a priori known cell type–specific markers. For seeds, such a bona fide cell type–specific data set is not yet available; therefore the researchers made use of spatial transcriptome technologies, Slide-seqV2. The Slide-seqV2 method is based on a combination of detecting RNAs at a spatial resolution of 10 *μ*m (Slide-seq) with single-cell trajectory analysis tools (Rodriques et al. 2019; Stickels et al. 2021). Using this methodology, it was revealed that the identified single-cell clusters spatially mapped to the cotyledons, root tip region, epidermis, seed coat, and provasculature. As such this dataset has the potential to function as foundation for future spatial transcriptome analyses in seeds. Important to consider when performing detailed transcriptome analyses is the material used for these investigations. Seeds are highly dynamic in their response to the environment, the changes they show over time (e.g. the differential expression when comparing TR vs nonruptured seeds from the same imbibition time-point) and on top of that show a large germination variability. With this we refer for example to the variation that occurs in a population of seeds harvested from a single plant (Mitchell et al. 2017). This variability likely contributes to the seed's role as a survival structure for seed plants and is nicely demonstrated by single-seed RNA-seq analyses performed on seeds during secondary dormancy induction (Krzyszton et al. 2022). Transcriptional variability is the lowest in seeds that have been exposed to the secondary dormancy, inducing stress, and is the largest in seeds that have recovered from this stress and have started germination (Fig. 2). Single-seed RNA-seq in combination with single-cell analyses might help to reveal the molecular basis of variability in seed pools at a cellular resolution.

## Epigenetic control of seed dormancy and germination

An important aspect of gene regulation is that controlled by epigenetic mechanisms. It has been reported that this mechanism, which includes DNA methylation of cytosines, histone, and in general chromatin modifications, controls important genes during seed development that affect dormancy and subsequent seed germination (Sato et al. 2021; Ding et al. 2022; Iwasaki et al. 2022; Sato and Köhler 2022). The function of many regulators of these processes had been described before using methods such as methylC-sequencing and ChIP-sequencing applied on mutants of these regulators. Often the respective mutants were used to test their effect on the above-mentioned seed development, seed dormancy, and germination processes leading to conclusions on the role of epigenetic mechanisms.

### Epigenetic regulation of seed maturation

Epigenetic control in seeds starts with the differential gene expression in the 2 gametes that together form the zygote and quicky develops into an embryo. This differential parental silencing distinguishes between maternal expressed genes (MEGs) and paternally expressed genes (PEGs). Two histone demethylases, *RELATIVE OF EARLY FLOWERING 6* (*REF6*) *and EARLY FLOWERING 6* (*ELF6*), control endosperm dormancy by activating the MEGs (Chen et al. 2020; Sato and Köhler 2022). A recent study analyzing natural variation for seed dormancy in Arabidopsis identified that VERNALIZATION5/VIN3-LIKE 3 (VEL3) maintains seed dormancy by establishing an epigenetic state in the central cell that primes the depth of primary seed dormancy, which is established later during seed maturation (Chen et al. 2023). VEL3 is required for deacetylation and the tri-methylation of lysine 27 on histone H3 (H3K27me3) established in the central cell. The authors propose that VEL3 maintains a repressive epigenetic state at ORE1 (OBSOLETE1) and other genes with a role in suppressing programmed cell death and inducing seed dormancy (Chen et al. 2023).

The role of epigenetic control during seed maturation included that of the major regulators of seed maturation LEC1, LEC2, FUS3, and ABI3 (Ding et al. 2022). Genetic studies subsequently have uncovered several loss-of-function mutations of chromatin-related genes that lead to ectopic expression of embryo-associated genes. These include mutants of the *HISTON 2B MONO-UBIQUINATION* genes *HUB1* and *HUB2*, resulting in reduced seed dormancy and, among others, downregulation of the dormancy gene *DOG1* (Liu et al. 2007). The proteins encoded by HUB1 and HUB2 most likely bind the polymerase II-associated factor 1 complex (PAF1C). This complex modulates the local structure of chromatin during transcription elongation and affects methylation of histone H3 at lysine 4 and 36 (H3K4me and H3K36me), respectively, which are activating epigenetic marks for transcription. Interestingly, the isolation and genetic analysis of additional factors associated with PAF1C, like REDUCED DORMANCY 2 in Arabidopsis, confirmed the important role of transcription elongation factors in seed maturation (Liu et al. 2007). Mutations in the *KRYPTONITE* (*KYP*)⁄*SU(VAR)3-9 HOMOLOG 4* (*SUVH4*) gene, encoding the histone methyltransferase for H3K9me2, cause increased seed dormancy. In accordance, KYP⁄SUVH4-overexpressing Arabidopsis plants show decreased dormancy (Zheng et al. 2012). This indicates that this repressive chromatin mark influences seed maturation. Potential direct targets of KYP⁄SUVH4 are the seed maturation genes *ABI3* and *DOG1*, which are upregulated in the *kyp-2* mutant.

DELAY OF GERMINATION-LIKE 4 (DOGL4) is a central factor mediating reserve accumulation in seeds without strongly affecting seed dormancy and other processes controlled by DOG1 (Sall et al. 2019). Zhu et al. (2018) described that the DNA demethylase ROS1 regulates the imprinting of *DOGL4*. *DOGL4* is expressed from the MEG and displays preferential methylation and suppression of the paternal allele. ROS1 negatively regulates imprinting by demethylating the paternal allele, preventing its hypermethylation and complete silencing. The dormancy effect of the *dogl4* mutant described by these authors is relatively weak compared with what is described for the *dog1* mutants (Sall et al. 2019). Epigenetic control of the dormancy master regulator *DOG1* was recently summarized by Ding et al. (2022).

The process of epigenetic silencing during germination involves the POLYCOMB REPRESSIVE COMPLEX 2 (PRC2), which plays a role in the repression of flowering in young seedlings by mediating H3K27me3. Mutants in FERTILIZATION INDEPENDENT ENDOSPERM, an essential component of the PRC2 complex, displayed genome-wide abolishment of H3K27me3 and exhibited increased seed dormancy and germination defects (Bouyer et al. 2011). Therefore, it was concluded that PRC2 is required for termination of the embryonic transcriptional program to promote the phase transition from embryo to autotrophic seedling. PRC2 sustains the balance between ABA and GA responsiveness via H3K27me3-mediated inhibition of positive ABA and negative GA regulators in maturing seeds. Moreover, the seed dormancy regulator DOG1 is repressed through PRC2-catalyzed H3K27- trimethylation at its locus (Bouyer et al. 2011).

Histone acetylation is another modification that plays a role in silencing of embryonic traits and activating genes associated with seedling establishment and growth. In Arabidopsis, HDA6 and HDA19 act redundantly to repress embryonic properties after germination (Tanaka et al. 2008). The function of *HDA9* is opposite to that of its homologous genes *HDA6* and *HDA19* (van Zanten et al. 2014). HDA9 negatively influences germination and is involved in the suppression of seedling traits in dry seeds. The HDA9 transcript is abundant in dry seeds and becomes reduced during imbibition in wild-type seeds.

### Epigenetic effects during seed germination

Dry seeds contain nuclei with reduced size and highly compacted chromatin; this phenomenon occurs during maturation and is therefore related to the acquisition of desiccation tolerance and seems independent from the dormancy state (van Zanten et al. 2012). Transcriptional reprogramming during germination requires repression of embryonic properties and activation of genes involved in the progression into photoautotrophic growth. Independent papers demonstrated roles for chromatin-remodeling factors and epigenetic signaling mechanisms in these processes. Seedlings of mutants lacking the CHD3 class SWI/SNF chromatin-remodeling factor PICKLE (PKL) display embryonic properties, that is, “pickle” roots, with a swollen and greenish distal root tip and accumulation of neutral lipid bodies (Ogas et al. 1999). PKL represses the expression of *ABI3* and *ABI5* in an ABA-dependent manner, as *pkl* mutants showed high *ABI3* and *ABI5* transcript levels after ABA treatment and hypersensitivity to ABA-mediated repression of germination (Perruc et al. 2007). The abundance of these epigenetic silencing marks was reduced in *pkl* mutants compared with the corresponding wild types, which was further enhanced by the application of ABA (Perruc et al. 2007). A direct role for PKL in determining levels of H3K27me3 at these repressed loci during germination was demonstrated (Zhang et al. 2012). Taken together, PKL controls ABA-mediated repression of germination by affecting the abundance of silencing epigenetic histone marks associated with the *ABI3* and *ABI5* loci. A detailed time course of germination using RNA seq showed that changes in gene expression and large-scale demethylation was observed toward the end of germination during the transitions from an embryo-like to a vegetative seedling state (Narsai et al. 2017).

## Translational regulation in seeds

As mentioned earlier, during seed maturation there is selective accumulation of mRNA also known as “stored mRNAs” or “long-lived mRNAs” (Ajtkhozhin et al. 1976; Ishibashi et al. 1990; Kuligowski et al. 1991). These stored mRNAs serve as a reservoir for rapid translation into proteins during germination (Ishibashi et al. 1990; Kuligowski et al. 1991; Rajjou et al. 2004; Sano et al. 2012). When nondormant Arabidopsis seeds were imbibed in a solution containing the transcriptional inhibitor α-amanitin, germination could still take place (Fig. 2). This indicated that the long-lived or stored mRNAs within the dry nondormant seed are sufficient to produce proteins required for successful germination. However, when Arabidopsis and rice seeds were imbibed in a solution containing the translational inhibitor cycloheximide, germination was blocked (Fig. 2; Rajjou et al. 2004; Sano et al. 2012, 2015). Therefore, the process of translation is crucial for germination to occur.

With the advent of technologies like ribosome profiling, studying the translational status of all mRNAs in seeds became possible. This technique employs sucrose-based fractions to separate mRNAs based on their association to no ribosomes (free mRNA), 1 ribosome (monosome), or multiple ribosomes (polysomes). mRNAs present in the polysome fraction most likely indicate active translation of those mRNAs into proteins. Using this technique, it was observed that dormant and nondormant seeds show differential association of mRNAs to polysomes (Layat et al. 2014). In the dormant embryos, negative regulators of germination seemed to be associated with polysomes, for example, transcription factors promoting ABA responses like ABI2 and LEA proteins, which should disappear during germination. Moreover, Bai et al. (2018) showed that translation was more abundant in AR Arabidopsis seeds than in dormant seeds at 24HAI. The addition of the transcriptional inhibitor cordycepin resulted in similar levels of translation in dormant and AR seeds, suggesting that transcriptional changes are required for the increased translational activity, which is seen in the nondormant seeds in control conditions. Based on this it was concluded that dormancy maintenance or alleviation is regulated at the transcriptional level (Bai et al. 2018).

Translational regulation, the control of the protein levels produced from its mRNA, is a highly efficient and reversible way of fine-tuning gene expression in response to developmental and environmental (Sablok et al. 2017; Chantarachot and Bailey-Serres 2018). To identify the translational efficiency of transcript one can determine the ratio of the mRNA abundance in the polysome fraction to the total RNA abundance of a given transcript between 2 timepoints or treatments (King and Gerber 2016). Using this approach, selective translation during seed maturation was reported (Bai et al. 2023). Among the translationally regulated genes are ABI2 and ABI5 (Finkelstein 1994; Finkelstein and Lynch 2000). It has also been reported that within the mature dry seed, most stored mRNAs are associated to monosomes and many are translationally upregulated upon imbibition. This indicates that these mRNAs are important for germination. mRNAs associated with polysomes in the dry seed are degraded during germination, suggesting that these are remnants from the maturation program and thus no longer needed for germination (Bai et al. 2020).

The first major study that implicated a role for translational regulation during germination came from Galland et al. (2014). In their study, they observed that differential accumulation of proteins occurred during early germination. The authors compared the transcript abundance, the rate of protein synthesis, and the protein abundance of 158 newly synthesized radiolabeled proteins during Arabidopsis seed germination. The protein abundance of these proteins, which included proteins involved in mRNA translation (10% of the identified proteins), Late Embryogenesis Abundant, and Heat Shock Proteins, correlated very well with the rate of protein synthesis and poorly with the amount of its transcript, thereby indicating that translational regulation occurs during germination for newly synthesized proteins (Galland et al. 2014). Another study performed in rice seeds showed that from the 20 proteins that were synthesized upon from the stored/long-lived mRNAs present dry seeds, only 12 of these were upregulated 2 days after imbibition, and the rest were upregulated 4 days after imbibition. Similarly, in Arabidopsis seeds, using ribosome profiling, Bai et al. (2017) showed that there is extensive sequence-specific translational regulation occurring during 2 shifts of germination, namely the hydration translational shift (HTS) and the germination translational shift (GTS). The HTS shift spans the transition from a dry seed to an imbibed seed, whereas the GTS marks the phase between 2 physiologically visible stages of germination, namely, TR and RP. The pools of mRNA that were translationally regulated were different in these 2 shifts, indicating independent mechanisms (Bai et al. 2017). The purpose of this regulation only in these 2 shifts of germination has not yet been established. However, during the HTS, the seed goes from a completely dry to a fully hydrated imbibed seed. Several metabolic processes must be kickstarted, and translation is initiated selectively from stored mRNA. This could be the reason why translational regulation occurs during the HTS, whereas during the GTS, the seed must make the all or nothing commitment to germinate and establish a seedling. This is a complex process based on several factors including a dynamically changing environment, which is why extensive translational regulation takes place during the GTS. Several mRNA features like upstream ORFs, transcript length, GC content, and secondary structures were correlated with an upregulation of translation.

### Mechanisms underlying translational control: the role of RNA binding proteins

The precise mechanism involved in the differential translation of mRNAs during germination is not known; however, RNA binding proteins (RBPs) are emerging as key players in the translational control of gene expression in plants (Hentze et al. 2018; Cho et al. 2019; Lou et al. 2020; Sajeev et al. 2022). RBPs are proteins that can bind target mRNAs through their RNA binding domain and regulate the fate and functions of their target mRNA (Lorković 2009; Li et al. 2014; Köster et al. 2017; Lou et al. 2020). Examples of these domains include RNA recognition motif, Pumilio/FBF domain, DEAD box domain, the K homology domain, and the Pentatricopeptide repeat motif.

RBPs have been implicated in seed development and performance (extensively reviewed by Lou et al. 2020). Some examples include RBPs like GLYCINE RICH PROTEIN 2 that can promote seed germination in stress conditions like low temperature via an ABA-independent pathway, whereas the overexpression of RNA DEAD box (RHs) helicases like RH9 and RH29 inhibit germination in salt conditions (Kim et al. 2005, 2007). APUM9, a conventional RBP from the PUMILIO family, plays a role in promoting germination in optimal conditions possibly by targeting ABA signaling genes for degradation (Xiang et al. 2014; Nyikó et al. 2019; Huang et al. 2021), and APUM5 plays a role in inhibiting seed germination in salt and osmotic stress (Huh and Paek 2014). The RBPs VARICOSE (VCS) and EXORIBONUCLEASE4 also play a role in seed dormancy and germination. The mutant seeds of *VCS* were less dormant, and those of *xrn-4* were more dormant (Basbouss-Serhal et al. 2017). Transcriptomic analyses of the *vcs* mutant seeds showed higher abundance of transcripts promoting germination like *WRKY60* and *WRKY63*, which are TFs that negatively regulate ABA signaling, while *xrn-4* seeds displayed higher transcript levels of genes involved in dormancy maintenance like ABA response elements *ABF3* and *ABP4* (Basbouss-Serhal et al. 2017). Interestingly, VCS and XRN are also components of higher-order RBP-mRNA complexes called processing bodies (P-bodies). These are membrane-less cytoplasmic granules that can transiently store or decay translationally repressed mRNAs (Maldonado-Bonilla 2014; Jang et al. 2020). Jang et al. (2019) reported that P-bodies control translation of certain mRNAs and are required for optimal seed to seedling transition.

Recently, the first repertoire of RBPs in germinating seeds that could provide potential candidates for future research was reported. This study employed an mRNA interactome capture approach that led to the identification of more than 200 high confidence RBPs present during the GTS (the phase between TR and RP) in seeds (GTS-RBPs) and 30 GTS-RBPs to be present specifically in seeds (Sajeev et al. 2022). Some of the identified RBPs were known to have a seed germination and dormancy phenotype. For instance, TUDOR SN1/2 has been shown to promote seed germination under salt stress by regulating the mRNA levels of a key GA biosynthesis enzyme, GA20ox3 (Maldonado-Bonilla 2014), and EIN2 has been reported to play a strong role in alleviating dormancy (Beaudoin et al. 2000; Ghassemian et al. 2000). This study also identified an RBP from the hyaluronan/mRNA, namely, AT5G47210, and showed that it plays a role in inhibiting germination under optimal conditions via an unknown mechanism (Sajeev et al. 2022).

## Concluding remarks and perspectives

In this review we provided an overview of the research that led to the current knowledge on the regulation of seed dormancy and germination in the past century. Still major questions remain to be solved. At the level of inducing and overcoming seed dormancy, ABA and DOG1 are major components. Although a lot is known about the ABA perception, the role of ABI3 in this has remained unclear. The same holds for the mechanism of the balance between ABA and GA. On the whole, genetic and natural variation studies in Arabidopsis have contributed significantly to our current knowledge on the regulation of seed dormancy and germination. Moreover, although not yet fully proven, there are strong indications that oxidative processes during AR change the dormancy state, which upon seed imbibition allow the seeds to germinate. The onset of germination itself seems more complicated. Nondormant seeds do not germinate if the environment is not optimal; however, the on/off switch for the decision to germinate has not yet been identified. Factors that could play a role in this are downstream genes of GA signaling, like TCPs and EXPAs; however, seeds contain many copies of these genes, and knocking out a single component generally only results in a small change in germination rate (Xu et al. 2020). Is this redundancy required to allow spreading the risk associated with germination in time and space, even in a single population, and does that mean that such an on/off switch does not exist? Or do we so far not have the insights or tools to identify this component? The use of single-cell analyses and mathematical modeling at the (sub)cellular scale might allow us to address this question. The component(s) that we are looking for may very well lie at the level of translation, as can be concluded from the strong translational control of seed germination at 2 critical physiological phases during seed germination (Bai et al. 2017). Employing techniques like interactome capture and individual-nucleotide resolution UV crosslinking and immunoprecipitation in seeds will advance our understanding and contribute to revealing the role of translational regulation in the control of seed germination. So far, technical difficulties have hampered advances in applying these high-tech protocols in seeds.

## Dive Curated Terms

The following phenotypic, genotypic, and functional terms are of significance to the work described in this paper:

VEL3 Gramene: AT2G18870
VEL3 Araport: AT2G18870

AFP2 Gramene: AT1G13740
AFP2 Araport: AT1G13740

BEE2 Gramene: AT4G36540
BEE2 Araport: AT4G36540

BES1 Gramene: AT1G19350
BES1 Araport: AT1G19350

ETR1 Gramene: AT1G66340
ETR1 Araport: AT1G66340

HDA6 Gramene: AT5G63110
HDA6 Araport: AT5G63110

IAA8 Gramene: AT2G22670
IAA8 Araport: AT2G22670

AHG1 Gramene: AT5G51760
AHG1 Araport: AT5G51760

AUX1 Gramene: AT2G38120
AUX1 Araport: AT2G38120

BIN2 Gramene: AT4G18710
BIN2 Araport: AT4G18710

EIN2 Gramene: AT5G03280
EIN2 Araport: AT5G03280

ABI2 Gramene: AT5G57050
ABI2 Araport: AT5G57050

RGL2 Gramene: AT3G03450
RGL2 Araport: AT3G03450

ABI4 Gramene: AT2G40220
ABI4 Araport: AT2G40220

ELF6 Gramene: At5g04240
ELF6 Araport: At5g04240
