# Correction to: A commitment for *life:* Decades of unraveling the molecular mechanisms behind seed dormancy and germination

**DOI:** 10.1093/plcell/koae165

**Published:** 2024-06-26

This is a correction to: Nikita Sajeev, Maarten Koornneef, Leónie Bentsink, A commitment for *life:* Decades of unraveling the molecular mechanisms behind seed dormancy and germination, *The Plant Cell*, Volume 36, Issue 5, May 2024, Pages 1358–1376, https://doi.org/10.1093/plcell/koad328.

Figure 1 has been corrected to swap the labels ‘primary dormant seed’ and ‘nondormant seed’.

